# Overactive IGF1/Insulin Receptors and NRASQ61R Mutation Drive Mechanisms of Resistance to Pazopanib and Define Rational Combination Strategies to Treat Synovial Sarcoma

**DOI:** 10.3390/cancers11030408

**Published:** 2019-03-22

**Authors:** Cinzia Lanzi, Laura Dal Bo, Enrica Favini, Monica Tortoreto, Giovanni Luca Beretta, Noemi Arrighetti, Nadia Zaffaroni, Giuliana Cassinelli

**Affiliations:** Department of Applied Research and Technological Development, Molecular Pharmacology Unit, Fondazione IRCCS Istituto Nazionale dei Tumori, Via Amadeo 42, 20133 Milan, Italy; laura.dalbo@istitutotumori.mi.it (L.D.B.); enrica.favini@istitutotumori.mi.it (E.F.); monica.tortoreto@istitutotumori.mi.it (M.T.); giovanni.beretta@istitutotumori.mi.it (G.L.B.); noemi.arrighetti@istitutotumori.mi.it (N.A.); nadia.zaffaroni@istitutotumori.mi.it (N.Z.)

**Keywords:** synovial sarcoma, pazopanib, drug resistance, BMS754807, trametinib, NRAS mutation, orthotopic sarcoma xenograft

## Abstract

Pazopanib is approved for treatment of advanced soft tissue sarcomas, but primary and secondary drug resistance limits its clinical utility. We investigated the molecular mechanisms mediating pazopanib resistance in human synovial sarcoma (SS) models. We found reduced cell sensitivity to pazopanib associated with inefficient inhibition of the two critical signaling nodes, AKT and ERKs, despite strong inhibition of the main drug target, PDGFRα. In the CME-1 cell line, overactivation of IGF1 and Insulin receptors (IGF1R/InsR) sustained AKT activation and pazopanib resistance, which was overcome by a combination treatment with the double IGF1R/InsR inhibitor BMS754807. In the highly pazopanib resistant MoJo cell line, NRASQ61R mutation sustained constitutive ERK activation. Transfection of the NRAS mutant in the pazopanib sensitive SYO-1 cell line increased the drug IC_50_. MoJo cells treatment with pazopanib in combination with the MEK inhibitor trametinib restored ERK inhibition, synergistically inhibited cell growth, and induced apoptosis. The combination significantly enhanced the antitumor efficacy against MoJo orthotopic xenograft abrogating growth in 38% of mice. These findings identified two different mechanisms of intrinsic pazopanib resistance in SS cells, supporting molecular/immunohistochemical profiling of tumor specimens as a valuable approach to selecting patients who may benefit from rational drug combinations.

## 1. Introduction

Synovial sarcoma (SS) comprises approximately 5–10% of all soft tissue sarcomas (STSs), primarily affecting adolescents and young adults. It is an aggressive tumor with a propensity to local recurrence and metastases that occur in about 50% of cases [1]. SS is characterized by a pathognomonic chromosomal translocation, t(X;18), which causes fusion of the SS18 gene with one of the SSX genes, SSX-1, -2, or rarely -4. Several lines of evidence indicate that the SS18–SSXs fusion genes, which encode proteins implicated in epigenetic dysregulation of gene expression, are the central genetic drivers in SS [2,3,4,5,6]. However, additional mutations detected with low frequency can alter the biology of SS, influencing the patients’ prognosis [7,8,9].

Surgery represents a mainstay for the control of local disease, in combination with (neo)adjuvant radiation therapy in high-risk patients. Cytotoxic chemotherapy with anthracycline and ifosfamide is generally applied as the frontline treatment for locally advanced and metastatic disease with response rates ranging between 25% and 40% [10,11]. SS is considered relatively more sensitive to chemotherapy than other STSs [12]. However, the overall patient survival of about 60% at 5 years has not substantially improved over the last few decades [13,14], and prognosis for advanced cases remains poor, highlighting the need for novel therapeutic approaches. SS18–SSX oncoproteins, which in principle represent ideal treatment targets, are currently undruggable. Nevertheless, based on new insights in SS biology, novel treatment options are emerging [10,11].

The small molecule multi-kinase inhibitor pazopanib is currently the only targeted drug approved for second-line treatment of STSs in the advanced setting. By targeting VEGF receptors, PDGF receptors, and to a lesser extent other kinases, pazopanib potentially inhibits both tumor cell growth and angiogenesis [15]. The precise mechanism of action of the drug in SS is not fully understood although evidence points to PDGFRα as a critical target in SS cells [16,17]. Despite an overall modest benefit, clinical trials demonstrated the potential of pazopanib to change the natural history of SS with patient outcomes characterized by high heterogeneity, including non-responders and long-term responders [11,18,19]. Similar to other clinically available targeted therapies, the occurrence of pazopanib resistance is frequent even in initially responsive patients, underlying the need to identify mechanisms of intrinsic and acquired drug resistance and to develop appropriate patient selection criteria and drug combination strategies to improve treatment outcome. Recent preclinical evidence indicates that resistance to pazopanib treatment can occur in SS cells through various mechanisms, including activating mutations of receptor tyrosine kinase (RTK) genes found with low frequency in SS, or by an adaptive cell kinome reprogramming following prolonged exposure to the drug [17,20,21,22].

Here, we investigated the mechanisms of pazopanib resistance occurring in SS cell lines and, based on the observed PDGFRα-bypassing signaling, we tested combination treatments aimed at overcoming drug resistance.

## 2. Results

### 2.1. Human SS Cell Lines Display Different Pazopanib Sensitivity Profiles In Vitro and In Vivo

**Figure 1:**
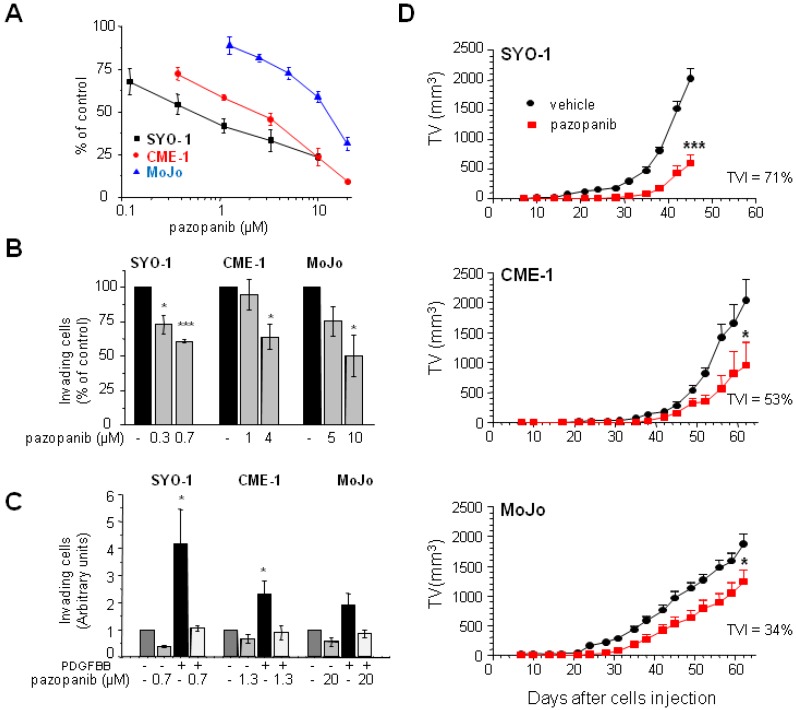
Antiproliferative, anti-invasive, and antitumor activity of pazopanib in synovial sarcoma (SS) models. (**A**) Cells were treated with increasing pazopanib concentrations one day (CME-1, SYO-1) or two days (MoJo) after plating. The drug antiproliferative activity was assessed by cell counting after 72 h (CME-1, SYO-1) or 96 h (MoJo). Average data ± SE from at least three independent experiments, performed in duplicate, are shown. (**B**,**C**) Cells pretreated for 24 h with pazopanib at the indicated concentrations were transferred onto Matrigel-coated Transwell chambers and incubated for an additional 24 h in the presence of complete medium (**B**) or in the absence of serum with or without PDGFBB (50 ng/mL) (**C**). Matrigel invasion is reported as percentage of invading cells ± SE (**B**) or as arbitrary units ± SE (**C**) from at least three independent experiments. (**D**) Inhibition of orthotopic SS xenograft growth in SCID mice. Pazopanib was administered by oral gavage at 100 mg/kg, qdx5/w, up to the end of the experiment, starting 3 days after i.m. tumor cell injection. Growth curves report the average tumor volumes (TV) in vehicle- and pazopanib-treated groups ± SE (6 mice/group). *, *p* < 0.05, ***, *p* < 0.001 vs. controls.

In our SS cell line panel, pazopanib IC_50_ varied from 0.6 µM to >15 µM without an apparent correlation with the type of SS18–SSX transcript expressed (Table S1 and Figure S1). For further studies, we focused on SYO-1, CME-1, and MoJo cells as models sensitive or moderately and highly resistant to the antiproliferative effect of pazopanib, respectively (Figure 1A). The same order of susceptibility to the drug inhibitory effect was evident in the Matrigel invasion assay in Figure 1B. Invasion stimulated by PDGF was abrogated by treatment with pazopanib, indicating the drug capability to hit PDGFR, a major target in SS cells [16,17]. Nonetheless, a different cell response to the growth factor was observed, with SYO-1 being the most responsive among the three cell lines (Figure 1C).

To assess the effect of pazopanib treatment on the SS models in vivo, we took advantage of the orthotopic tumorigenicity of the three cell lines previously demonstrated in severe combined immunodeficient SCID mice [23]. Mice harboring i.m. injected tumor cells were administered daily with the drug. Growth curves indicated a tumor growth delay induced by treatment. At the end of the experiment, tumor volume inhibition percentages (TVI%) of 71, 53, and 34 for SYO-1, CME-1, and MoJo, respectively, compared to vehicle treatment, confirmed the different susceptibility of the SS xenografts to the drug (Figure 1D).

Overall, these experiments suggested a reduced dependence on PDGFR signaling in the pazopanib resistant SSs and the potential contribution of other cell intrinsic factors driving drug resistance.

### 2.2. Reduced Cell Sensitivity to Pazopanib Is Associated with Lowered Inhibition of AKT or ERKs

**Figure 2:**
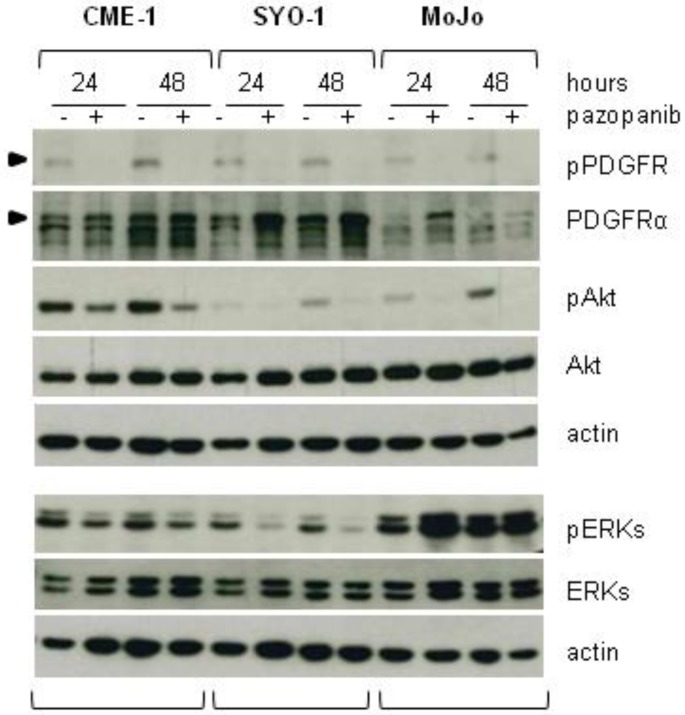
Effects of pazopanib on PDGFRα activation and downstream pathways in SS cell lines. Cells were treated the day after seeding with solvent or pazopanib at a concentration around two times the IC_50_ (5 µM CME-1, 1.3 µM SYO-1, 20 µM MoJo cells), for 24 h and 48 h. Then, cells were lysed and processed for Western blot analysis with the indicated antibodies to detect activation and expression levels of PDGFRα, AKT, and ERKs. Samples from the same experiment were analyzed on two separate filters, each with its loading control (actin).

SSs are characterized by high levels of PDGF receptors [24], which are well known pazopanib targets [15]. Specifically, PDGFRα appears to be uniquely overexpressed in SS relative to other sarcomas [16]. We compared the effects of pazopanib on receptor activation and signaling in our three cell lines (Figure 2). Western blot analysis showed lower levels of PDGFRα in MoJo cells, although tyrosine phosphorylation, indicative of receptor activation, was comparable and completely abolished by pazopanib in all three cell lines. In contrast, the drug effects on downstream signaling were different in the three cell lines. Whereas AKT activation was strongly reduced by pazopanib in both SYO-1 and MoJo cells, only a partial reduction was achieved in CME-1 cells. ERK activation was strongly inhibited in the most sensitive SYO-1, moderately inhibited in CME-1, and unaffected or even enhanced (after 24 h of treatment) in the pazopanib resistant MoJo cells.

As ERK1/2 activation by pazopanib has been associated with dysregulation of the autophagic-flux, which could ultimately influence the cellular outcome [25,26,27,28], we examined the effect of the RTK inhibitor on the autophagic process over time. In SYO-1 and CME-1 cells, pazopanib did not substantially affect the levels of the lipidated form of LC3 (LC3II) present on the autophagosome membranes. In contrast, the drug induced a marked accumulation of LC3II, suggestive of impaired autophagic flux [29], in MoJo cells (Figure S2A). Consistently, in these cells, the levels of p62, a substrate specifically degraded during autophagy, and those of LAMP2, a lysosomal structural protein, were upregulated by treatment and remained high for up to 72 h. In SYO-1 and CME-1 cells, p62 and LAMP2 levels were not altered by treatment (Figure S2B).

Taken together, these data indicated that cell sensitivity to pazopanib was associated with a substantial inhibition of the critical signaling nodes AKT and ERKs and, in the highly resistant MoJo cells, the persistent ERK activation was associated with impairment of the autophagic flux.

### 2.3. Inhibition of Overactive IGF1R/InsR Overcomes Pazopanib Resistance in CME-1 Cells

**Figure 3:**
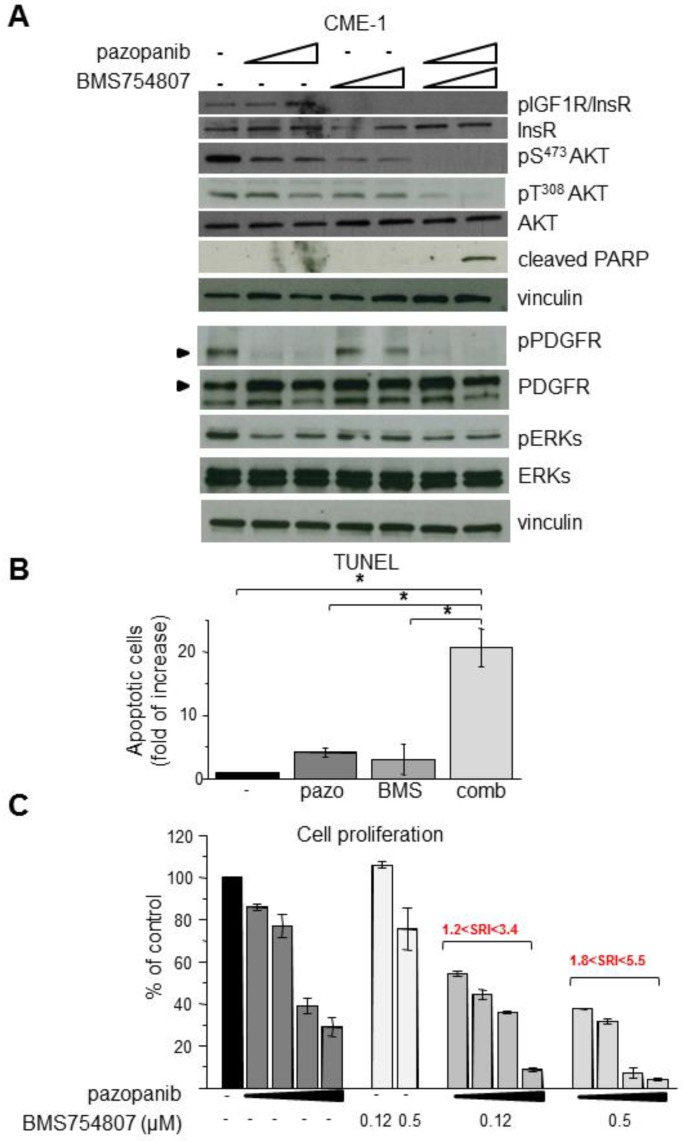
Propaptotic and synergistic antiproliferative activity of combination treatment with pazopanib and the IGF1R/InsR inhibitor BMS754807 in CME-1 cells. (**A**) Cells were exposed to pazopanib (2.5, 5 µM) followed 24 h later by BMS754807 (0.12, 0.5 µM) as single agents or in combination. After 24 h, cells were lysed and processed for Western blot analysis with the indicated antibodies. (**B**) Cells were exposed to 5 µM pazopanib followed 24 h later by 0.5 µM BMS754807 alone or in combination. After 48 h, cells were processed for detection of apoptosis by TUNEL assay. Columns and bars represent mean values ± SD from two independent experiments. * *p* < 0.05. (**C**) After 24 h of exposure to pazopanib (1.25, 2.5, 5, and 10 µM), cells were treated with BMS754807 at 0.12 or 0.5 µM as indicated and 48 h later counted by a Coulter Counter. Drug interaction was evaluated by calculation of the synergistic ratio index (SRI), values > 1 indicate synergy. Columns and bars represent mean values ± SD from one representative experiment out of two, performed in duplicate.

A sustained activation of AKT and ERKs in resistant cells upon pazopanib treatment could be supported by upstream signaling pathways independent of PDGFR. We previously showed that CME-1 cells display a constitutive activation of IGF1R and high levels of activation of both IGF1R and InsR in complete growth medium [23]. Because either PDGFR or IGF1R can contribute to sustaining AKT activation in SS cells [16], we hypothesized that the combination with BMS754807, a dual IGF1R/InsR inhibitor, could overcome the moderate pazopanib resistance of CME-1 cells. In fact, Western blot analysis of cells subjected to the combined treatment showed a complete abrogation of AKT activating phosphorylations of both T^308^ and S^473^ coupled with PARP cleavage, suggesting induction of apoptosis (Figure 3A). pERK was reduced by both drugs but not abrogated by the combination, suggesting that the IGF1R/InsR-AKT pathway is the most relevant in the pazopanib resistant phenotype of CME-1 cells. The apoptotic death of cells treated with both drugs, as well as the synergistic drug interaction, were further confirmed by TUNEL and proliferation assays (Figure 3B,C).

### 2.4. Aberrant Activation of NRAS/ERKs/MKP3 Pathway in Pazopanib Highly Resistant MoJo Cells

**Figure 4:**
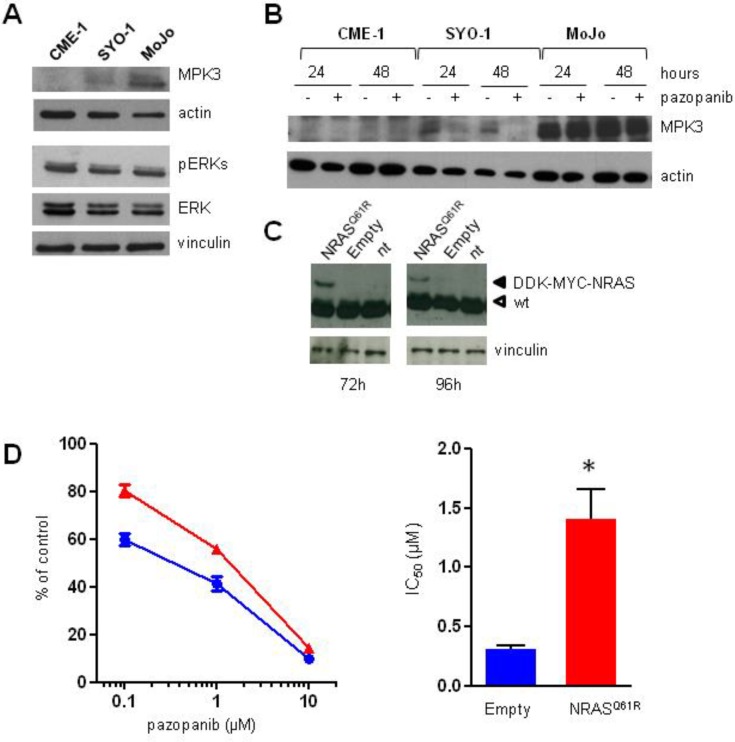
Aberrant expression of ERK phosphatase MKP3 in pazopanib resistant NRASQ61R mutated MoJo cells, and reduced pazopanib sensitivity of SYO-1 cells expressing exogenous NRASQ61R. SS cells were analyzed for MKP3 expression by Western blotting in basal conditions (**A**) and after exposure to pazopanib at around two-fold IC_50_ (5 µM CME-1, 1.3 µM SYO-1, 20 µM MoJo cells) (**B**). (**C**) SYO-1 cells were transiently transfected with empty or NRASQ61R expression vectors. Levels of RAS proteins were analyzed by Western blotting at 72 h and 96 h after transfection (DDK–MYC–NRAS, tagged mutated NRas; wt, wild type Ras). (**D**) Twenty-four hours after transfection, SYO-1 cells were exposed to pazopanib for 72 h to assess the drug antiproliferative effects by cell counting. On the left are dose-response curves from three independent experiments. On the right are the IC_50_ values (drug concentrations producing 50% inhibition). Columns ± bars: IC_50_ mean values ± SD * *p* < 0.05.

MAPK pathways are controlled downstream by dual-specificity MAP kinase phosphatases (MKPs) [30]. To address the role of ERKs activation in pazopanib resistant MoJo cells, we analyzed expression of the ERK-specific phosphatase MKP3 (DUSP6). Indeed, enhanced ERK activation has been recently associated with in vitro acquired SS resistance to pazopanib as a consequence of MKP3 downregulation [21]. Conversely, we found MKP3 markedly overexpressed in MoJo compared to SYO-1 cells and not detectable in CME-1 cells (Figure 4A). Moreover, whereas MKP3 levels were reduced by drug treatment in SYO-1 cells, no substantial modulation was induced in MoJo cells (Figure 4B). These data suggested that a different mechanism involving the ERK pathway could be implicated in the intrinsically resistant MoJo cells.

MoJo cells were originally found to harbor the NRAS Q61R mutation (K.B. Jones and M. Ladanyi, personal communication), further confirmed by cDNA sequencing (Figure S3). Since the constitutive activation of the RAS/ERK pathway could account for MKP3 overexpression [31] as well as for the lack of ERK inhibition by pazopanib in MoJo cells, we exogenously expressed NRASQ61R in SYO-1 cells to assess whether the RAS mutation affected cell sensitivity to the drug. Following transient transfection, expression of the NRAS mutant protein was maintained for at least 96 h (Figure 4C). A significant reduction of cell sensitivity to pazopanib was observed in a 72 h-cell proliferation assay (96 h transfection), with a nearly 4-fold increase of the IC_50_ in NRASQ61R- as compared to mock-transfected SYO-1 cells (Figure 4D).

### 2.5. Inhibition of MEK1/2 Overcomes Pazopanib Resistance in NRASQ61R Mutant MoJo Cells

**Figure 5:**
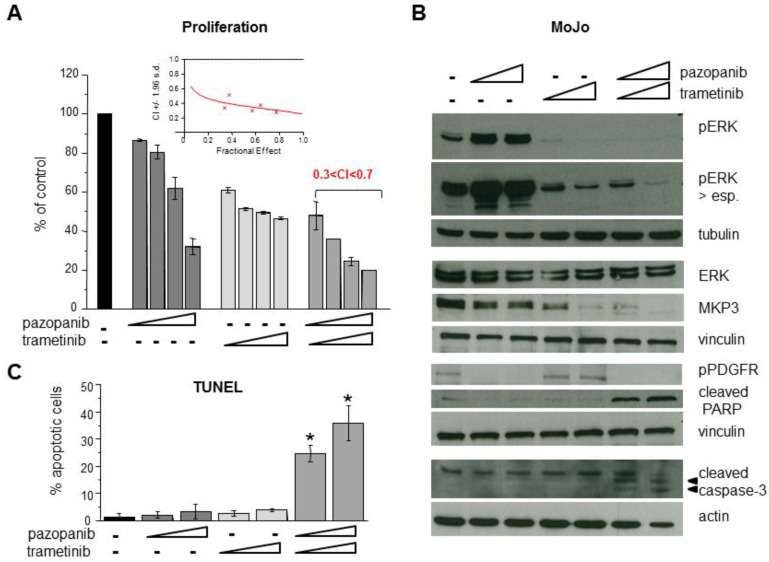
Antiproliferative and proapoptotic effects of the combination of pazopanib with the MEK1/2 inhibitor trametinib in pazopanib resistant MoJo cells. (**A**) Cells were exposed to pazopanib (2.5, 5, 10, 20 µM) or trametinib (0.5, 1, 2, 4 nM) as single drugs or in simultaneous combination. The antiproliferative effect was assayed by cell counting 96 h later. Drug interaction was evaluated by Combination Index (CI), values < 1 indicate synergy. Inset: representative quantitative diagnostic plot (CI vs. fractional effect). (**B**,**C**), Cells were treated with pazopanib (10, 20 µM) or trametinib (2, 4 nM) alone or in combination for 48 h. Then, cells were processed by Western blot analysis to assess the effects on PDGFR and ERK pathway activation and cleavage of apoptosis related proteins (PARP and caspase-3) (**B**), or for apoptosis detection by TUNEL assay (**C**). In (**B**), samples from the same experiment were analyzed in separate filters, each with its loading control (tubulin, vinculin, or actin). In (**A**,**C**), columns and bars represent mean percentage values ± SD from two independent experiments. * *p* < 0.05 vs. either control or single drug treatment.

The constitutive NRASQ61R-driven ERK activation, and its contribution to pazopanib resistance, suggested that inhibition of the ERK pathway could enhance MoJo cell response to pazopanib. We thereby tested a combination treatment of pazopanib with the MEK1/2 inhibitor trametinib. Consistent with the NRASQ61R mutant expression [32], MoJo cells showed high sensitivity to the trametinib antiproliferative effect (IC_50_ = 1.6 ± 0.4 nM in MoJo cells vs. > 1 μM in SYO-1 cells). Figure 5A shows that trametinib dose-dependently inhibited ERK activation and reduced MPK3 levels, confirming dependence of the phosphatase expression on ERKs. The combination of the two drugs abrogated ERK stimulation by pazopanib and induced cleavage of caspase-3 and PARP. Apoptosis was also confirmed by TUNEL assay (Figure 5B) and a drug synergistic interaction was evidenced by proliferation assay in the combination treated cells (Figure 5C). Pazopanib-induced upregulation of p62 was also prevented by the combination treatment (Figure S4), supporting a protective role of autophagy in MoJo cell response to the drug.

**Figure 6:**
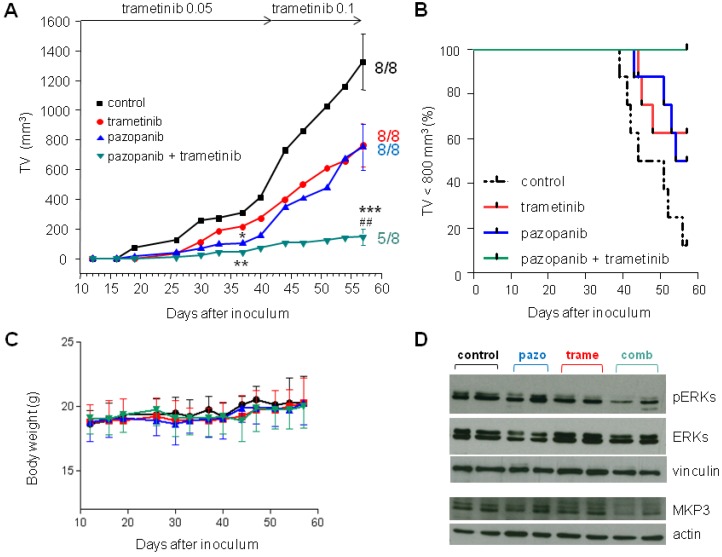
Antitumor activity of pazopanib in combination with trametinib against MoJo orthotopic tumor xenografts in mice. Twelve days after i.m. injection of MoJo cells, mice were randomized in four groups (eight animals each) and treated with vehicle (control), pazopanib (100 mg/kg), or trametinib (0.05 mg/kg and then 0.1 mg/kg as indicated by arrows) as single agents or in combination. (**A**) Tumor growth curves during the treatment period. Each point is the mean tumor volume (TV) in the group. Bars represent SE at treatment end. * *p* < 0.05, ** *p* < 0.01, *** *p* < 0.001 vs. controls, ## *p* < 0.01 vs. both single drug treatments, by Student’s *t*-test. (**B**) Kaplan Meier curves for the four groups considering tumor size exceeding 800 mm^3^ as an endpoint. *p* = 0.003 by log-rank test. (**C**) Body weights of mice during the treatment period. Each point is the mean weight of mice in each group. Error bars represent SD (Change percentages at the end of treatments: 8.7 ± 9.3, 7.9 ± 6.5, 8.4 ± 10.3, 4.6 ± 6.6 in control, pazopanib, trametinib, and combination groups, respectively). (**D**) Pharmacodymamic effect on ERKs and MKP3 in tumors from mice receiving pazopanib (pazo), trametinib (trame), or the combination (comb). At day 57, mice were sacrificed and two tumors/groups were removed and processed for Western blot analysis of ERK activation and MPK3 expression.

We further tested the combination of the two drugs in the mouse orthotopic model. Mice inoculated with tumor cells were orally treated with vehicle, with pazopanib (100 mg/kg, qdx5d/w), or trametinib (0.05 mg/kg, qdx5d/w) alone or in combination. The low dose of the MEK inhibitor was chosen based on the high sensitivity in vitro of MoJo cells to the drug. After 4 weeks of treatments, 37 days after tumor cells inoculation, all tumors (8/8) were measurable in control mice. In the groups receiving trametinib, pazopanib, or the combination, measurable tumors were 7/8, 4/8, and 4/8, respectively. The two drugs singly administrated attenuated tumor growth, although the mean tumor volume was statistically different from that of controls only in the group receiving pazopanib (*p* < 0.05). The combination treatment resulted in a significantly enhanced inhibition of tumor growth (*p* < 0.01 vs. vehicle) (Figure 6A).

Given the very low dosage used of the MEK inhibitor, we explored the possibility to further enhance the antitumor effect of the combination treatment by increasing the trametinib dose to 0.1 mg/kg starting from day 42. At the end of the experiment (day 57), all mice receiving a single drug treatment had measurable tumors, and a similar tumor volume inhibition (TVI) (43%) was achieved in both groups barely approximating significance (*p* = 0.059 trametinib, *p* = 0.053 pazopanib). In contrast, the drug combination maintained tumor growth control (89% TVI, *p* < 0.001 vs. controls, *p* < 0.01 vs. single pazopanib or trametinib) and no measurable tumor was found in 3 out of 8 (38%) mice. Kaplan Meier analysis considering tumor size exceeding 800 mm^3^ as an endpoint showed a highly significant difference between curves (*p* = 0.001) (Figure 6A,B). No obvious signs of toxicity were observed in drug treated mice and, consistently, body weights were not significantly affected by treatments (Figure 6C). Western blot analysis of tumor samples harvested at sacrifice confirmed the enhanced inhibition of ERK activation and a parallel reduced expression of MKP3 in combination treated mice (Figure 6D).

## 3. Discussion

Identification of somatic alterations in human tumors is increasingly evidencing the co-occurrence of changes affecting more than one signaling pathways, which can functionally synergize and contribute to drug resistance [33,34]. This study identified signaling pathways bypassing PDGFRα, the main SS cellular target of the multi-tyrosine kinase inhibitor pazopanib, in human SS cell lines resistant to the drug. Overactivation of IGF1R/InsR in CME-1 and NRAS Q61R mutation in MoJo cells were found to be associated with pazopanib intrinsic resistance, which was overcome by combining the drug with inhibitors of IGF1R/InsR or MEK1/2, respectively. Pazopanib is currently used in STS after failure of standard chemotherapy. Its peculiar activity in SS patients has been underlined in several clinical studies [10,11,18,19,35,36,37]. Nevertheless, the drug’s therapeutic efficacy remains unsatisfactory and biomarkers of sensitivity/resistance, as well as new rationally designed approaches, are needed to improve treatment outcome.

Our current findings support efficient inhibition of the AKT and ERKs signaling nodes as a determinant in SS cell sensitivity to pazopanib. AKT activation has been reported in clinical SS specimens and constitutive activation of the PI3K–AKT pathway is thought to occur in most cases through RTK mediated autocrine/paracrine loops [24]. Overexpression/activation of PDGFRα is a peculiar feature of SS among other sarcomas [16,24,38]. The pazopanib-sensitive cell line SYO-1 used in our study, similar to another SS cell line, HS-SY-11, has been demonstrated to be dependent on the PDGFRα signaling pathway for proliferation [39]. In other SS cell lines, activation of alternative RTKs such as Met and ALK has been associated with resistance to pazopanib [17,20,22,39]. Our study reveals the activation of IGF1R and InsR as an additional RTK-mediated mechanism of pazopanib resistance, bypassing PDGFR and sustaining PI3K–AKT signaling activation. Combination of pazopanib with the IGF1R/InsR dual inhibitor BMS754807 suppressed AKT activation and induced apoptosis in CME-1 cells in agreement with a critical role of the pathway in SS cell growth and survival [23,40]. Drug resistance driven by co-expression of RTKs and their ligands can represent one aspect of the tumor cell aggressive phenotype. Insulin-like growth factor 2 is transcriptionally induced by SS18–SSX oncoproteins and expression of IGF1R correlates with SS aggressiveness [41,42,43]. Similarly, co-expression of hepatocyte growth factor and the Met receptor, described as associated with Met dependency and pazopanib resistance in Yamato-SS cells [17,20,39], correlates with poor prognosis in SS patients [8,20]. In this regard, Naka and colleagues have hypothesized that expression and activation of RTKs in SS reflect the cellular origin (more or less immature mesenchymal stem cell) that provides a permissive background for the oncogenic driver SS18–SSX [20,39]. ALK is an additional RTK potentially able to influence the SS biological behavior. A truncated functional form of the receptor has been recently identified in the pazopanib resistant ASKA–SS cells. Moreover, ALK rearrangement has been found in one out of 43 SS patient specimens [17].

Studies reporting analyses at the genomic level have documented additional infrequent genetic alterations in SS clinical samples co-occurring with the chromosomal translocation generating the SS18–SSX fusion oncogenes. Among these, mutations in cancer-associated genes, although rare, may account for the disease’s variable clinical behavior and response to therapy [9,44,45]. RAS missense mutations in SS have been reported in two previous studies. Oda et al. identified HRAS mutations at codon G12 in three out of 49 cases [8], whereas Vlanterie et al. found KRAS G12 mutations in one out of 41 cases [9]. Here, we showed that the NRAS Q61R mutation present in the MoJo SS cell line impaired the pazopanib antiproliferative effect. In fact, exogenous expression of the NRAS mutant in the sensitive SYO-1 cells significantly increased the drug IC_50_. Activating RAS mutants have been implicated in the pathogenesis of several neoplastic diseases [46]. The three H, K, and N RAS genes encode highly related GTPases that bind signaling effectors mediating RTK activated pathways. Amino acid substitution at G12, G13, and Q61 residues impede the GTPase’s return to a GDP-bound inactive state so that the mutant RAS proteins are constitutively active even in the absence of RTK signaling. NRAS mutants are capable of inducing constitutive activation of the RAF–MEK–ERK and the PI3K–AKT pathways [46]. The high sensitivity of MoJo cells to the antiproliferative effect of trametinib, a highly specific MEK1/2 inhibitor, suggested a prominent function of the RAF–MEK–ERK pathway in cell growth regulation. Moreover, the high levels of the ERK-specific MKP3 phosphatase, which is transcriptionally induced by ERK, reflected an enhanced negative feedback required to avoid growth arrest or cell death caused by excessive ERK activation [47]. It is noteworthy that, in apparent contrast with our data, reduced MKP3 levels, also enhancing ERK activation, have been associated with in vitro acquired pazopanib resistance [21]. Convergence on ERK pathway activation of different mechanisms of intrinsic and acquired resistance underlines the relevance of this signaling axis in the modulation of SS cell sensitivity to the drug. The persistent RAS–ERK signaling in MoJo cells is also consistent with the heightened state of autophagy, reflected by LC3II accumulation, as described in other RAS mutant cancer cells [46,48]. Pazopanib not only was unable to inhibit the constitutive RAS signaling but, as previously described in other resistant cancer cell lines [26], further enhanced ERK activation and the autophagic process. Prevention of these paradoxical effects accounted for the synergistic interaction of pazopanib with trametinib in MoJo cells. In fact, the two drugs in combination promoted apoptosis in tumor cells and strongly potentiated the antitumor activity against the pazopanib resistant SS xenograft. Notably, both RAS mutation and MKP3 expression have been found to predict sensitivity to the MEK inhibitor in several solid tumor and hematological malignancy cell lines [49].

Trametinib has been approved for treatment of advanced BRAF-mutant melanoma, non-small cell lung cancer, anaplastic thyroid carcinoma [50,51,52], and multiple clinical studies are examining trametinib-based combinations in a variety of tumor types including melanoma and other tumors with NRAS-ERK pathway activation [53] (https://clinicaltrials.gov). The combination of trametinib with pazopanib has shown promising preclinical activity in thyroid cancer and renal cell carcinoma models [54,55]. In a phase I study in unselected STS patients [56], the combination was reasonably well tolerated, in agreement with our findings in the SS xenograft, suggesting that the MEK inhibitor could be safely combined with pazopanib for treatment of patients with NRAS mutated SS.

## 4. Materials and Methods

### 4.1. Cell Lines and Culture Conditions

The human SS cell lines SYO-1 [57] and MoJo [58], provided by K.B. Jones (University of Utah, Salt Lake City, UT, USA), were maintained for routine cell culture in DMEM medium (Lonza, Verviers, Belgium) supplemented with non-essential amino acids and 10% or 20% fetal bovine serum (FBS), respectively. CME-1 cells [59,60], provided by M. Pierotti (Istituto FIRC di Oncologia Molecolare, Milan, Italy), were cultured in RPMI medium (Lonza) with 10% FBS. The expression of the SS18–SSX fusion products in these cell lines was confirmed and periodically controlled by RT-PCR and Western blot analyses as described [23,61].

Additional human SS cell lines used in this study include: 1273/99, donated by O. Larsson (Karolinska Institute, Stockholm, Sweden), which were cultured in Ham’s F12 (Lonza) with 20% FBS [62]; Yamato-SS and Aska-SS, originally established by Naka N et al. [63] and provided by Y.M.H. Versleijen-Jonkers (Radboud University Medical Center, Nijmegen, The Netherlands), which were maintained in DMEM supplemented with 10% and 20% FBS, respectively.

### 4.2. Drugs

Pazopanib was purchased from SelleckChem (Houston, TX, USA), BMS754807 from Active Biochemicals (Wanchai, Hong Kong, China), and trametinib from Santa Cruz Biotechnology (Santa Cruz, CA, USA). For in vitro studies, drugs were dissolved in DMSO and further diluted in cell culture medium (0.25–0.5% DMSO final concentration).

### 4.3. Cellular Studies

Cells were treated with drugs, one or two days after plating, depending on growth rate. Drug antiproliferative effects were assessed after 72 h or 96 h by cell counting using a Coulter Counter (Beckman Coulter, Luton, UK). In combination experiments, CME-1 cells were exposed to pazopanib and, 24 h later, treated with BMS754807; MoJo cells were simultaneously treated with pazopanib and tramenitib. To evaluate drug interactions, the synergistic ratio index (SRI) was calculated as described by Kern et al. [64]. According to this method, the expected value of cell survival is defined as the product of the survival observed for the two drugs alone:

Expected Survival = Survival Drug A × Survival Drug B

and the SRI is calculated as the ratio of expected and observed survival:

SRI = Expected Survival/Observed Survival

SRI > 1 indicates synergy while SR ≤ 1 indicates the absence of synergy/additive effect.

Additionally, drug interaction was analyzed and graphed with the use of the CalcuSyn 26 Software (Biosoft, Cambridge, UK) according to Chou–Talalay [65]. By this method, a combination index (CI) value = 1 indicates an additive effect, CI < 1, synergy, and CI > 1, antagonism.

For invasion assay, cells were pretreated with the indicated drug in complete medium for 24 h, then harvested and transferred to the upper chamber of 24-well Transwell plates (Costa, Corning Inc., Corning, NY, USA) previously coated with Growth Factor Reduced Matrigel (BD Biosciences, San Jose, CA, USA) (6–8 × 10^5^ cells/filter, according to the spontaneous invasive ability). The same drug concentration used for cell pretreatment was added to both the upper and lower chambers. For experiments performed in the presence of PDGFBB (Sigma, St. Louis, MO, USA), the growth factor was added to the lower chamber at 50 ng/mL in serum-free medium. After 24 h of incubation, cells that invaded Matrigel were stained with sulforhodamine B as described [66] and counted.

Apoptosis was assessed in floating and adherent cells by TUNEL (Terminal deoxynucleotidyl transferase dUTP Nick End Labeling) assay (Roche, Mannheim, Germany) according to the manufacturer’s instructions. Samples were analyzed by the flow cytometer Accuri C6 or, alternatively, FACSCelesta (Becton Dickinson, San Jose, CA, USA).

### 4.4. Analysis of NRAS Mutational Status

Exponentially growing MoJo and SYO-1 cells were harvested and processed for RNA extraction. cDNA was obtained using the High Capacity Reverse Transcription Kit according to the manufacturer protocol (Applied Biosystems, Foster City, CA, USA) and PCR amplified (USB FideliTaq PCR Master Mix (2X) Affymetrics, Santa Clara, CA, USA) for NRAS using the following primers: NRAS-Forward 5′-CAGGTGGTGTTGGGAAAAGC-3′; NRAS-Reverse 5′-CTCGCTTAATCTGCTCCCTGT-3′. NRAS DNA fragments were purified (PureLink PCR Purification Kit, Thermo Fisher Scientific, Waltham, MA, USA) and sequenced (Eurofins Genomics, Ebersberg, Germany).

### 4.5. NRASQ61R Transfection

SYO-1 cells were transiently transfected with 0.1 μg/mL of pCMV6-Entry empty vector or NRAS(Q61R) Myc-DDK-tagged plasmid (Origene Technologies, Rockville, MD, USA) in Lipofectamine 3000 (Invitrogen, Carlsbad, CA, USA) the day after seeding. The CCA>CGA substitution at NRAS codon 61 in the plasmid was previously confirmed by sequencing (Eurofins Genomics) using VP1.5 and XL39 primers (Origene Technologies). Cell lysates were analyzed for NRAS expression by Western blotting at 72 h and 96 h after transfection. To assess sensitivity to pazopanib of NRAS transfectants, cells were exposed to increasing drug concentrations 24 h after transfection and counted 72 h later.

### 4.6. Western Blotting

Cells were seeded in complete medium and treated with drugs at the indicated concentrations 24 h or 48 h later. Total protein extraction and Western blotting with the indicated primary antibodies were performed as previously described in detail [67]. Lysates from frozen tumors were analogously processed after pulverization by the Mikro-Dismembrator II (B. Brown Biotech International, Melsungen, Germany).

### 4.7. Antibodies

The following antibodies were used for Western blot analyses.

- Mouse monoclonal: anti-PKBα/Akt from BD Transduction (Lexington, KY, USA); anti-tubulin and anti-vinculin from Sigma; anti-LAMP2 and anti MPK3/DUSP6 from Abcam (Cambridge, UK); anti-pan-Ras from Calbiochem (San Diego, CA, USA); anti-p62 from MBL (Woburn, MA, USA).
- Rabbit monoclonal: anti-phospho-IGF1-IRβ (Tyr1135/1136)/InsRβ (Tyr1150/1151) (19H7) and anti-phospho-PDGFRα (Tyr849)/PDGFRβ (Tyr857) from Cell Signaling Technology (Beverly, MA, USA).
- Rabbit polyclonal: anti-SYT (H-80) and anti-InsRβ (C-19) from Santa Cruz Biotechnology. Anti-cleaved PARP (Asp214), anti-phospho-p44/42 MAPK (Thr202/Tyr204), anti-p44/42 MAPK, anti-phospho-Akt (Ser473), anti-cleaved Caspase-3 (Asp175), and anti-LC3B from Cell Signaling Technology; anti-PDGFRα from Upstate Biotechnology (Lake Placid, NY, USA); anti-pan-Akt phosphoT308 antibody from Abcam; anti-actin from Sigma.

### 4.8. In Vivo Studies

All in vivo experiments were authorized by the Italian Ministry of Health (project N° 55/2016-PR, 21 January 2016) and were performed in accordance with the EU Directive 2010/63/EU for animal experiments, internal institutional guidelines, and international policies [68]. Experiments were carried out using 8 week-old female SCID mice (Charles River, Calco, Italy) randomized in groups of 6–8 mice, each bearing one i.m. tumor. In preliminary experiments, performed to evaluate in vivo sensitivity to pazopanib treatment, exponentially growing cells were injected orthotopically (i.m.) (5 × 10^6^ SYO-1, 20 × 10^6^ CME-1 and MoJo) in the right leg of SCID mice and treatments started 3 days after tumor cells injection. Pazopanib, dissolved in 0.5% methylcellulose was administered by gavage at 100 mg/kg daily for 5 consecutive days per week (qdx5d/w).

In the combination experiment, treatments started 12 days after MoJo cells injection (20 × 10^6^ i.m.). Trametinib, dissolved in DMSO and further diluted in PBS, was administered by oral gavage at 0.05 mg/kg qdx5d/w for 4 weeks and then at 0.1 mg/kg for an additional 3 weeks. Pazopanib, prepared as above, was administered two hours after trametinib treatment by oral gavage at 100 mg/kg qdx5d/w for 7 weeks. Control mice were treated with the drug vehicle.

For in vivo pharmacodynamic evaluation, at day 57, 2 h after the last drug administration, the animals were sacrificed and tumors (2–3/group) were resected and snap frozen in liquid nitrogen before processing for Western blotting.

The efficacy of treatments was assessed as tumor volume inhibition percentage (TVI%) calculated according to the formula:

TVI% = 100 − (mean TV treated/mean TV control × 100).

where TV is tumor volume.

Drug tolerability was evaluated as body weight loss.

### 4.9. Statistical Analyses

Two-tailed Student’s *t*-test was used to compare two sets of data. Log rank (Mantel-Cox) test was used to compare Kaplan Meier curves. Analyses were performed using the GraphPad Prism software, version 4.0 (GraphPad Prism Inc., San Diego, CA, USA). *p* values < 0.05 were considered as statistically significant.

## 5. Conclusions

Although pazopanib has been recognized as a potent antiangiogenic agent, this study supports the concept that tumor cell-based mechanisms can contribute to intrinsic drug resistance in SS. We described two mechanisms, based on IGF1R/InsR and NRAS mutation-driven ERK pathway activation, which add to other previously described mechanisms able to bypass the pazopanib-sensitive PDGFR pathway. Importantly, these alterations co-expressed with the SS18–SSX oncoproteins are actionable and their targeting can potentially overcome drug resistance. Taken together, these data underline the relevance of molecular and immunohistochemical interrogation for resistance-associated targets to improve SS treatment outcome by patient-specific pazopanib-based combination therapies. Moreover, implementation of the use of patient-derived ex vivo systems (e.g., primary cell cultures, 3D systems) is also expected to provide an opportunity to discover new mechanisms of drug resistance and improve personalized treatments [69,70].

## Supplementary Materials

The following are available online at https://www.mdpi.com/2072-6694/11/3/408/s1, Table S1: Antiproliferative activity of pazopanib in human synovial sarcoma cell lines, Figure S1: Expression of SS18–SSX fusion genes and proteins in SS cell lines, Figure S2: Effect of pazopanib on autophagic-lysosomal pathway in SS cell lines, Figure S3: Sequence analysis of NRAS gene mutation at codon 61 in MoJo cells, Figure S4: Effects of the combination of pazopanib with the MEK1/2 inhibitor trametinib on autophagic flux and apoptosis in MoJo cells.
