# Supplementary material for: Overactive IGF1/Insulin Receptors and NRASQ61R Mutation Drive Mechanisms of Resistance to Pazopanib and Define Rational Combination Strategies to Treat Synovial Sarcoma
Source: Cancers (Basel). 2019 Mar 22;11(3):408. doi: 10.3390/cancers11030408 (PMC6468361; doi:10.3390/cancers11030408)
Supplement: Supplementary file 1 [file cancers-11-00408-s001.zip › Figure.S2_R.pdf]

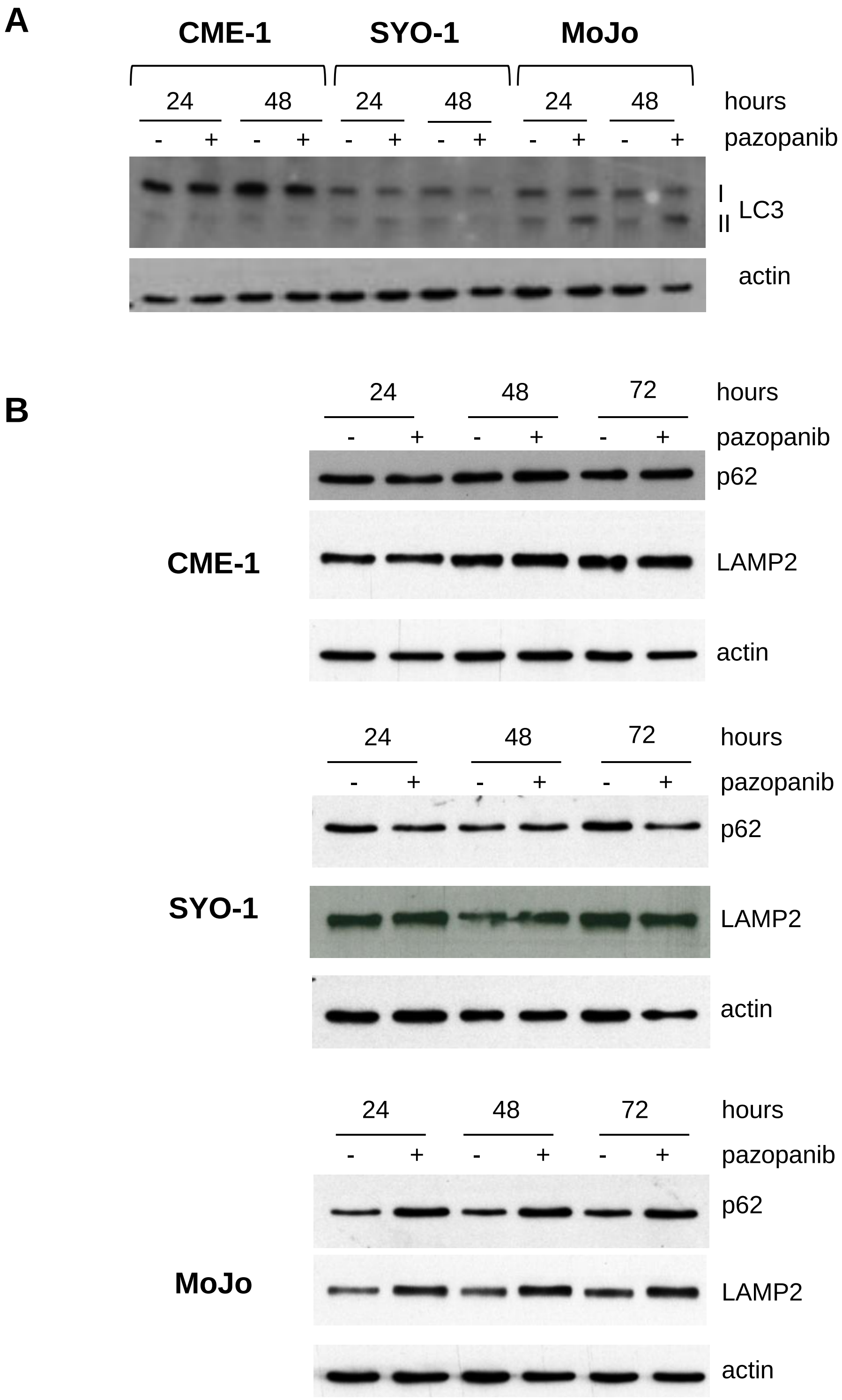

Figure S2. Effect of pazopanib on autophagic-lysosomal pathway in SS cell lines. Cells were exposed to solvent or pazopanib (5 µM CME-1 cells, 1.3 µM SYO-1 cells, 20 µM MoJo cells) for the indicated times and then lysed and processed for western blot analysis to monitor overtime the effect on levels of autophagy-related proteins. (**A**) Levels of LC3I and its lipidated form LC3II. (**B**) Levels of p62 and lysosomal protein LAMP2.
