# Supplementary material for: Overactive IGF1/Insulin Receptors and NRASQ61R Mutation Drive Mechanisms of Resistance to Pazopanib and Define Rational Combination Strategies to Treat Synovial Sarcoma
Source: Cancers (Basel). 2019 Mar 22;11(3):408. doi: 10.3390/cancers11030408 (PMC6468361; doi:10.3390/cancers11030408)
Supplement: Supplementary file 1 [file cancers-11-00408-s001.zip › Figure.S3.pdf]

Sequence obtained using NRAS-Forward primer

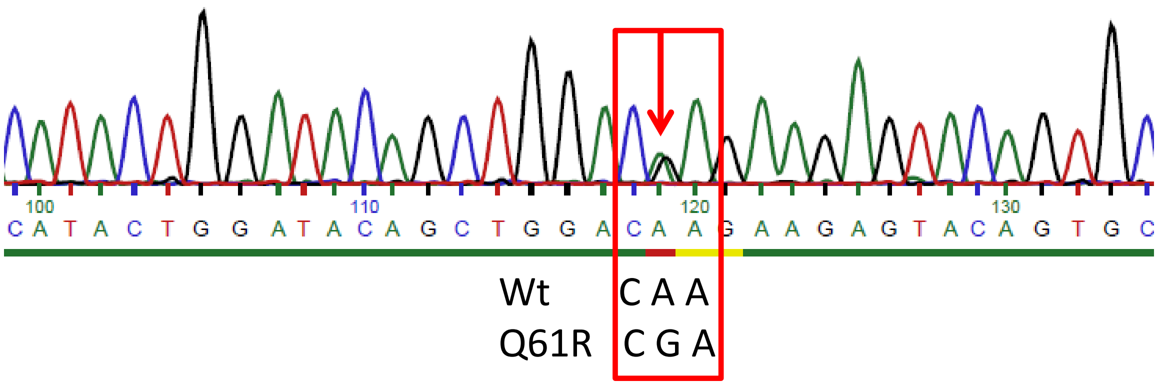

Sequence obtained using NRAS-Reverse primer

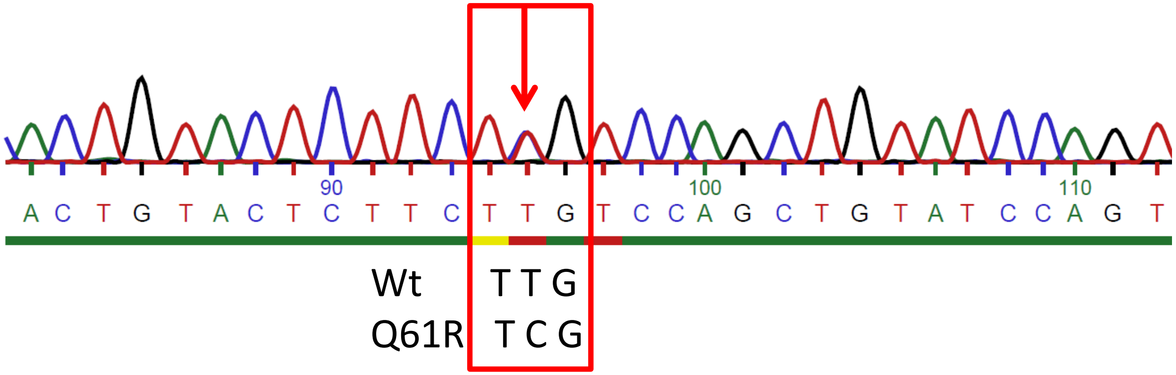

Figure S3. Sequence analysis of NRAS gene mutation at codon 61 in MoJo cells. cDNA was obtained from total RNA extracted from exponentially growing cells. Specific primers were used to amplify the cDNA sequence encompassing NRAS codon 61. Sequence analysis was carried out on purified DNA fragments. Sequence chromatograms obtained with NRAS forward and reverse primers are shown.
