# Supplementary material for: Overactive IGF1/Insulin Receptors and NRASQ61R Mutation Drive Mechanisms of Resistance to Pazopanib and Define Rational Combination Strategies to Treat Synovial Sarcoma
Source: Cancers (Basel). 2019 Mar 22;11(3):408. doi: 10.3390/cancers11030408 (PMC6468361; doi:10.3390/cancers11030408)
Supplement: Supplementary file 1 [file cancers-11-00408-s001.zip › Figure S4_R.pdf]

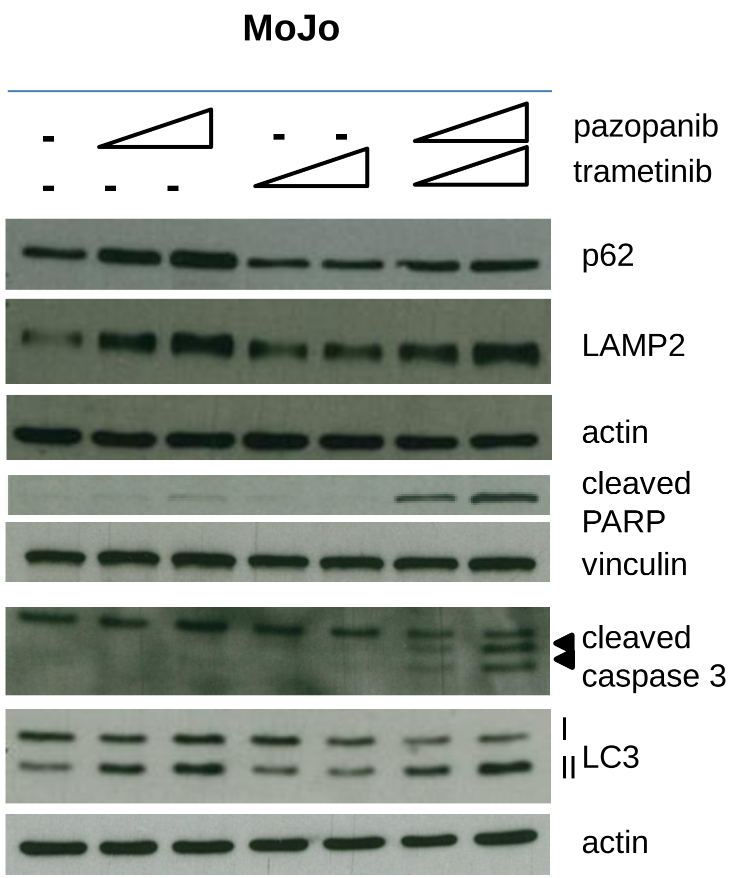


**Figure S4.** Effects of the combination of pazopanib with the MEK1/2 inhibitor trametinib on autophagic flux and apoptosis in MoJo cells. Cells were treated with pazopanib (10 and 20 µM) or trametinib (2 and 4 nM) alone or in combination for 24h. Then, cells were processed for western blot analysis using the indicated antibodies to assess drug treatment effects on the levels of autophagy-related proteins (p62, LAMP2 and LC3) and processing of apoptosis-related proteins (PARP, caspase-3). Samples were analyzed in three separate filters, each with its loading control (actin or vinculin).
