# Supplementary material for: Overactive IGF1/Insulin Receptors and NRASQ61R Mutation Drive Mechanisms of Resistance to Pazopanib and Define Rational Combination Strategies to Treat Synovial Sarcoma
Source: Cancers (Basel). 2019 Mar 22;11(3):408. doi: 10.3390/cancers11030408 (PMC6468361; doi:10.3390/cancers11030408)
Supplement: Supplementary file 1 [file cancers-11-00408-s001.zip › Table S1.pdf]

Table S1

Antiproliferative activity of pazopanib in human synovial sarcoma cell lines

Cell line	Fusion transcript	pazopanib IC_{50} [µM] [a]	hours of treatment
SYO-1	SS18-SSX2	0.6± 0.1	72
CME-1	SS18-SSX2	3 ± 0.29	72
MoJo	SS18-SSX1	13 ± 3	96
Aska-SS	SS18-SSX1	5 ± 1.2	96
Yamato-SS	SS18-SSX1	> 15	96
1273/99	SS18-SSX2	> 15	96

[a] IC_{50}, drug concentration producing 50% inhibition, determined by cell counting after 72h or 96h of treatment, according to the cells' growth rate.
