# Supplementary material for: Overactive IGF1/Insulin Receptors and NRASQ61R Mutation Drive Mechanisms of Resistance to Pazopanib and Define Rational Combination Strategies to Treat Synovial Sarcoma
Source: Cancers (Basel). 2019 Mar 22;11(3):408. doi: 10.3390/cancers11030408 (PMC6468361; doi:10.3390/cancers11030408)
Supplement: Supplementary file 1 [file cancers-11-00408-s001.zip › Figure.S1.pdf]

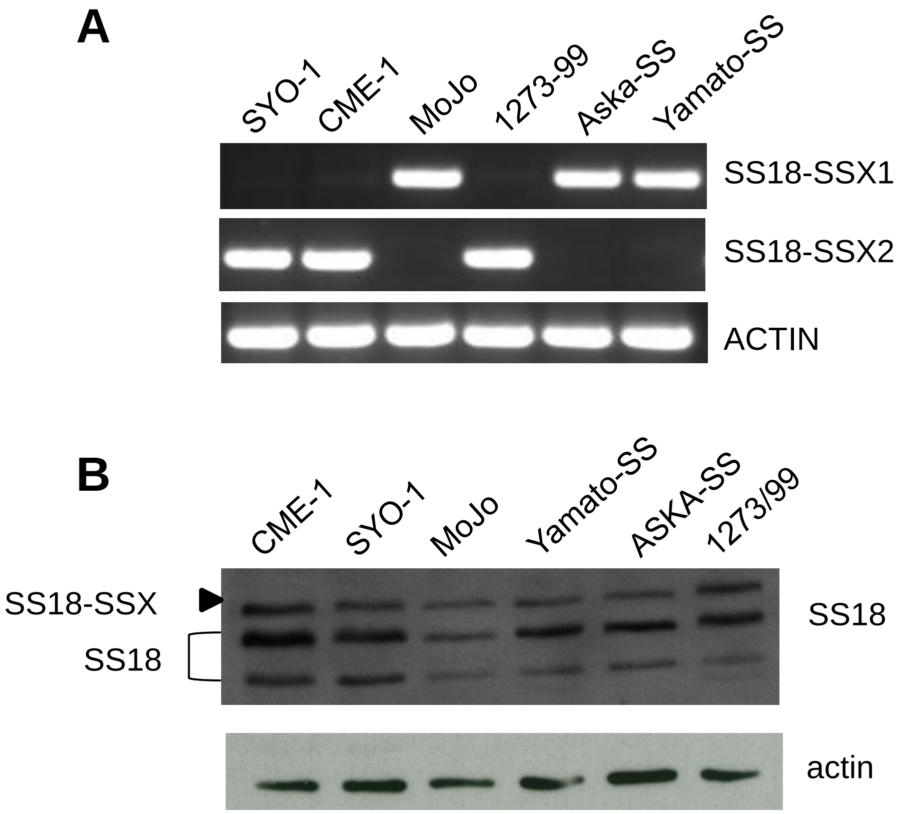

Figure S1. Expression of SS18-SSX fusion genes and proteins in SS cell lines. **A**) Expression of SS18-SSX transcripts detected by RT-PCR with specific sets of primers for the SS18-SSX1 and SS18-SSX2 chimeric genes. **B**) Expression of SS18-SSX fusion proteins detected by western blotting using anti-SS18 antibody. Actin serves as housekeeping gene and loading control.
